# Molecular Markers Distinguishing Early‐Stage Mycosis Fungoides From Atopic Dermatitis Skin Lesions

**DOI:** 10.1111/exd.70240

**Published:** 2026-03-26

**Authors:** Brandon D. Ng, Conor Whelan, Natalia Alkon, Agata Kurowski, Constanze Jonak, Patrick M. Brunner

**Affiliations:** ^1^ Department of Dermatology, Icahn School of Medicine New York USA; ^2^ Department of Dermatology Medical University of Vienna Vienna Austria

**Keywords:** atopic dermatitis, CTCL, mycosis fungoides, scRNA‐seq

## Abstract

Mycosis fungoides (MF) is the most common type of primary cutaneous T‐cell lymphoma, a disease characterized by malignant T cells that home to the skin. In early stages, clinical presentation is often indistinguishable from benign chronic inflammatory skin diseases such as atopic dermatitis (AD), posing a challenge for proper diagnosis and treatment. Previous studies have established that MF is characterized by the expansion of a single T‐cell clone, whereas benign skin conditions are polyclonal in nature. In this study, we aimed to use single‐cell RNA sequencing data to detect distinct transcriptomic features of early‐stage MF in comparison to AD skin. In early‐stage MF, we observed gene expression differences in cells of both the stroma and the immune system, with keratinocytes exhibiting increased interferon response and proliferation (*STAT1*, *ICAM1*, *HLA‐DRA*, *GJB2*), while fibroblasts displayed tumour‐associated programs (*CXCL2*, *TNFAIP6*, *CEBPD*). Myeloid cells exhibited expression of immunomodulatory genes (*RUNX3*, *DDIT4*, *IL4I1*), and malignant T‐cells expressed exhaustion‐associated markers (*CXCL13*, *SOCS3*, *F2R*, *ETV1*), as opposed to AD and healthy control samples. Thus, our results provide a novel insight into the immune‐stroma crosstalk in the tissue microenvironment of early‐stage MF vs. AD skin lesions.

**Abbreviations**
AD: Atopic dermatitis
CTCL: Cutaneous T‐cell lymphoma
esMF: Early‐stage mycosis fungoides
MF: Mycosis fungoides
scRNA‐seq: Single‐cell RNA sequencing
TCR: T‐cell receptor
Treg: Regulatory T cell

## 1 Introduction

Mycosis fungoides (MF) is a potentially life‐threatening disease of malignant T‐cells that home to and proliferate in the skin. The disease usually occurs in patients > 50 years of age but paediatric patients can also be affected [1]. As part of a larger disease spectrum of cutaneous T‐cell lymphomas (CTCLs), early‐stage mycosis fungoides (esMF) is often clinically and histopathologically indistinguishable from patients with chronic benign inflammatory skin diseases such as atopic dermatitis (AD) [2], as both diseases often present as sharply demarcated, erythematous, scaling patches or plaques. While AD is generally a Th2‐mediated disease characterized particularly by overexpression of IL‐13 in the skin [3, 4], esMF has been described to exhibit a more Th1‐skewed phenotype characterized by IFN‐γ production [5]. MF lesions have been known to be dominated by the expansion of a single T‐cell clone [6, 7, 8, 9], compared to the polyclonal nature of T‐cells contributing to AD. Nonetheless, the diagnosis of esMF can be challenging, with a median of three years from the appearance of the first lesion until a correct diagnosis is made [10]. Thus, additional techniques and insights are needed to distinguish esMF from AD in a timely manner. We aimed to determine molecular differences between esMF and AD lesions, to enhance our understanding of gene expression in these two diseases, and to facilitate future discovery of better diagnostic biomarkers. Using single‐cell RNA (scRNA) and T‐cell receptor (TCR) sequencing, we compared esMF and AD biopsies, with healthy control skin serving as baseline. Our analyses revealed gene expression differences in both stromal and immune cell types. esMF exhibited a generally immunomodulatory signature from stromal and myeloid cells, revealing several mediators that might aid in a better understanding of esMF lesion development in comparison to AD.

## 2 Methods

### 2.1 Sample Acquisition, Processing and Analysis

Data of this manuscript are based on a cohort previously published by Alkon et al. [11]. Patients were included after obtaining written informed consent of an IRB‐approved protocol (Table S1). All patients had previous histopathological assessment, and only samples with an expanded T‐cell clone were assigned a diagnosis of CTCL. Lesional skin punch biopsies were processed for scRNA‐seq using the Chromium Single Cell Controller and Single Cell 5 Library & Gel Bead Kit v1.1 (10X Genomics, Pleasanton, CA), according to the manufacturer's protocol. TCR‐αβ sequences were enriched from cDNA using the respective reagents, following the instructions of the VDJ Kit workflow by 10X Genomics. Sequencing was performed using the Illumina NovaSeq platform and the 150 bp paired‐end configuration. FASTQ files were processed with Cell Ranger v6.1.2 (10X Genomics) with human genome GrCh38. The subsequent processed files were analysed using Seurat (v5.3.0) and BiocManager (v1.30.26). The scDblFinder package (v1.22.0) was implemented to exclude doublets from each sample individually, using 200 nearestneighbours. Cells with > 10% mitochondrial genes and either < 500 or > 3000–7000 genes (nFeature_RNA) were filtered out on each sample individually. After filtering, reciprocal PCA (rPCA) was used to integrate all samples together.

Ambient RNA contamination from keratinocytes or fibroblasts was present in our samples, leading to expression of keratinocyte‐specific or fibroblast‐specific genes in other cell types. Following an initial round of Louvain clustering, we assigned cell lineages and in all cell populations we observed bimodal populations for many genes specific to keratinocyte and fibroblasts. Using this bimodal distribution, we performed gene filtering with the following cutoffs: expression level > 2 for the genes *S100A13*, *S100A16*; expression level > 2.5 for the genes *S100A14*, *GSTP1*, *GSN*; expression level > 3 for the genes *LGALS7*, *DSP*, *S100A4*, *S100A10*, *KRT2*, *LY6D*, *KRT6B*, *KRT6C*, *FABP5*, *KRT17*, *AQP3*, *CXCL14*; expression level > 3.5 for the genes *S100A2*, *SFN*, *DMKN* and *PERP*; and expression level > 4 for the genes *KRT1*, *KRT10*, *KRT5*, *KRT14*, *S100A6*, *S100A7*, *S100A8*, *S100A9*, *KRT6A*, *KRT16*, *KRTDAP*, *LGALS7B*. Following this filtering, we re‐performed rPCA and Louvain clustering with “FindClusters”. Additional subclustering was performed with “FindSubclusters”.

Differentially expressed gene (DEG) lists were obtained with the “FindMarkers” command, using the Wilcoxon Rank Sum test, Bonferroni correction for multiple comparisons, and setting adjusted *p*‐value to < 0.05, log‐fold change > 0.5, and default min.pct value of 0.1. Ribosomal and mitochondrial genes were filtered out from the generated DEG lists.

## 3 Results

### 3.1 Analysis of Single‐Cell RNA and T‐Cell Receptor Sequencing Data From Early‐Stage Mycosis Fungoides in Comparison to Atopic Dermatitis and Healthy Control Skin

**FIGURE 1:**
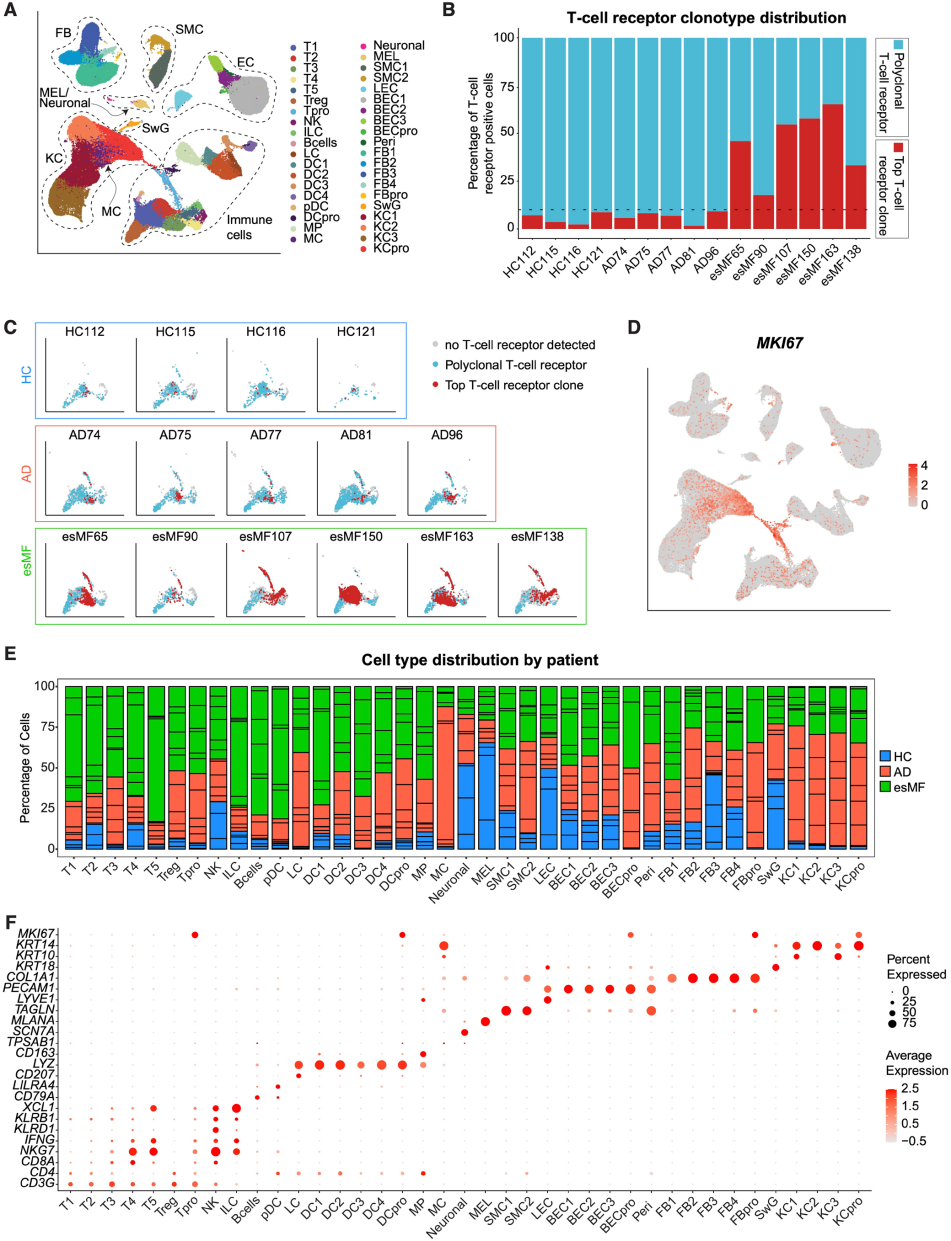
Single‐cell RNA and T‐cell receptor sequencing map comparing early‐stage mycosis fungoides (esMF) with atopic dermatitis (AD) and healthy control (HC) skin biopsies. (A) UMAP projection of all 108 487 cells used in this analysis post quality control and filtering, with 39 distinct cell populations annotated. (B) Frequencies of the single most abundant T‐cell clone per sample (red) in comparison to the residual polyclonal T‐cell receptor (TCR) + population (blue). Dashed line represents the cutoff between a monoclonal (> 10%) and overall polyclonal (< 10% top clone) process. (C) UMAP projection mapping the top abundant T‐cell clone (red) per sample within the larger T‐ and NK‐cell cluster (blue and grey). (D) UMAP projection displaying *MKI67* expression across all cells. Expression levels are colour‐coded and overlaid onto UMAP plots. Intensity of red colour represents high expression levels. (E) Barplot showing distribution of cell frequencies across patients and cell clusters. (F) Dotplot of canonical gene expression in each of the 39 cell types annotated. Circle size represents the percentage of cells expressing the specific marker within a cluster. Colouring denotes scaled average expression levels within each cluster. *UMAP: Uniform Manifold Approximation and Projection, T: T‐cells, Tpro: Proliferating T cells, NK: Natural killer cells, ILC: Innate lymphoid cells, Bcells: B cells, LC: Langerhans cells, DC: Dendritic cells, pDC: Plasmacytoid dendritic cells, MP: Macrophages, MC: Mast cells, Neronal: Neuronal cells, MEL: Melanocytes, SMC: Smooth muscle cells, LEC: Lymphatic endothelial cells, BEC: Blood endothelial cells, Peri: Pericytes, FB: Fibroblasts, SwG: Sweat gland cells KC: Keratinocytes*.

To characterize differences between early‐stage mycosis fungoides (esMF) and atopic dermatitis (AD) skin lesions, we integrated six esMF biopsies with five AD and four HC samples (Table S1) from a previously published dataset [11]. scRNAseq integration and unsupervised clustering yielded 108 487 cells in 39 distinct cell clusters (Figure 1A, Table S2). All esMF samples were characterized by the presence of a single dominant T‐cell clone exhibiting > 10% abundance of the total T cell receptor (TCR) + clone pool (Figure 1B,C), which is a hallmark characteristic of MF [12]. By contrast, top clone frequencies of all AD and HC samples were < 10% (Figure 1B,C), consistent with an overall benign, polyclonal T‐cell repertoire. We observed substantial proliferation in T‐cell, dendritic cell (DC), keratinocyte (KC), fibroblast (FB), and blood endothelial cell populations, as evidenced by *MKI67* expression (Figure 1D).

When assessing the distribution of cell counts across cell populations, we observed that in many T‐cell clusters, esMF samples contributed to the majority of total cells, whereas KC were dominated by AD samples (Figure 1E; Table S2 and Table S3). Canonical marker expression of all cell clusters is depicted in Figure 1F. Notably, B‐cells and plasmacytoid dendritic cells (pDCs) had incomplete resolution of clustering, resulting in some contamination of *CD79A*+ B‐cells in the *LILRA4+* pDC cluster (Figure 1F). Additionally, mast cells were unable to fully resolve from the KC populations. Thus, neither B‐cells, pDCs, nor mast cells were included for subsequent analysis in this study.

### 3.2 Differentially Expressed Genes in Both esMF and AD Patients Are Most Pronounced in Keratinocyte and Fibroblast Populations

**FIGURE 2:**
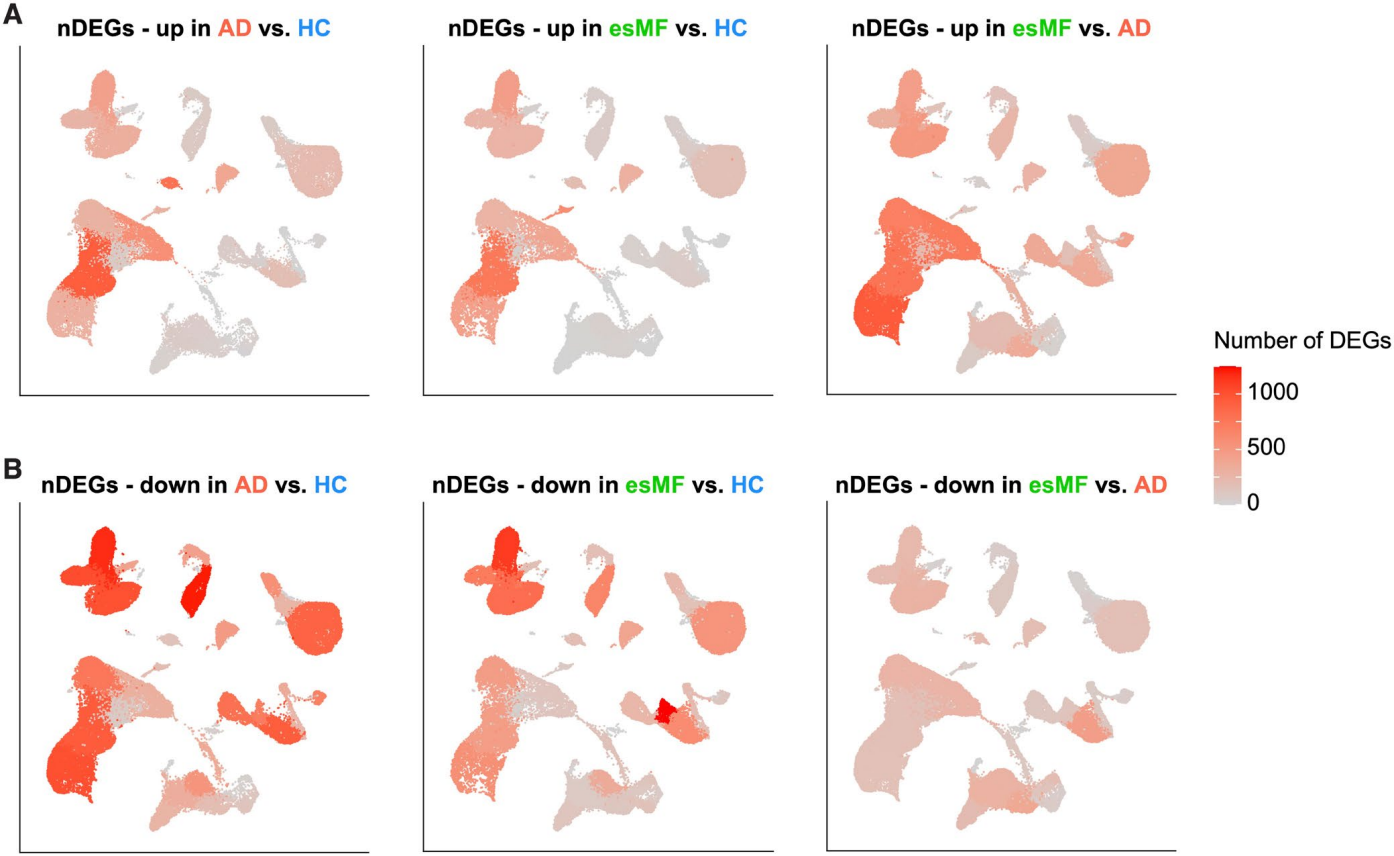
Number of differentially expressed genes (DEGs) found between groups per cell cluster. (A) UMAP projection showing the number of DEGs upregulated between indicated pairwise comparisons for each cell cluster. (B) UMAP projection showing the number of DEGs downregulated between indicated pairwise comparisons for each cell cluster. Expression levels are colour‐coded and overlaid onto UMAP plots. Intensity of red colour represents numbers of DEGs.

We next performed pairwise comparisons to calculate differentially expressed genes (DEGs) between individual sample groups, showing high levels of transcriptomic dysregulation both in esMF and AD (Figure 2A,B; Table S4). Among genes upregulated in both disease groups compared to HC skin, we found highest numbers within KC, followed by FB (Figure 2A). KC were also the populations with highest numbers of DEGs between esMF and AD samples. Among downregulated DEGs, stromal cells showed highest numbers, with additional dysregulation in myeloid cells and T cells. Taken together, highest numbers of DEGs in any comparison were found primarily in KC.

### 3.3 Keratinocytes in esMF Exhibit an Inflammatory Signature Dominated by Interferon Signalling and Antigen Presentation

**FIGURE 3:**
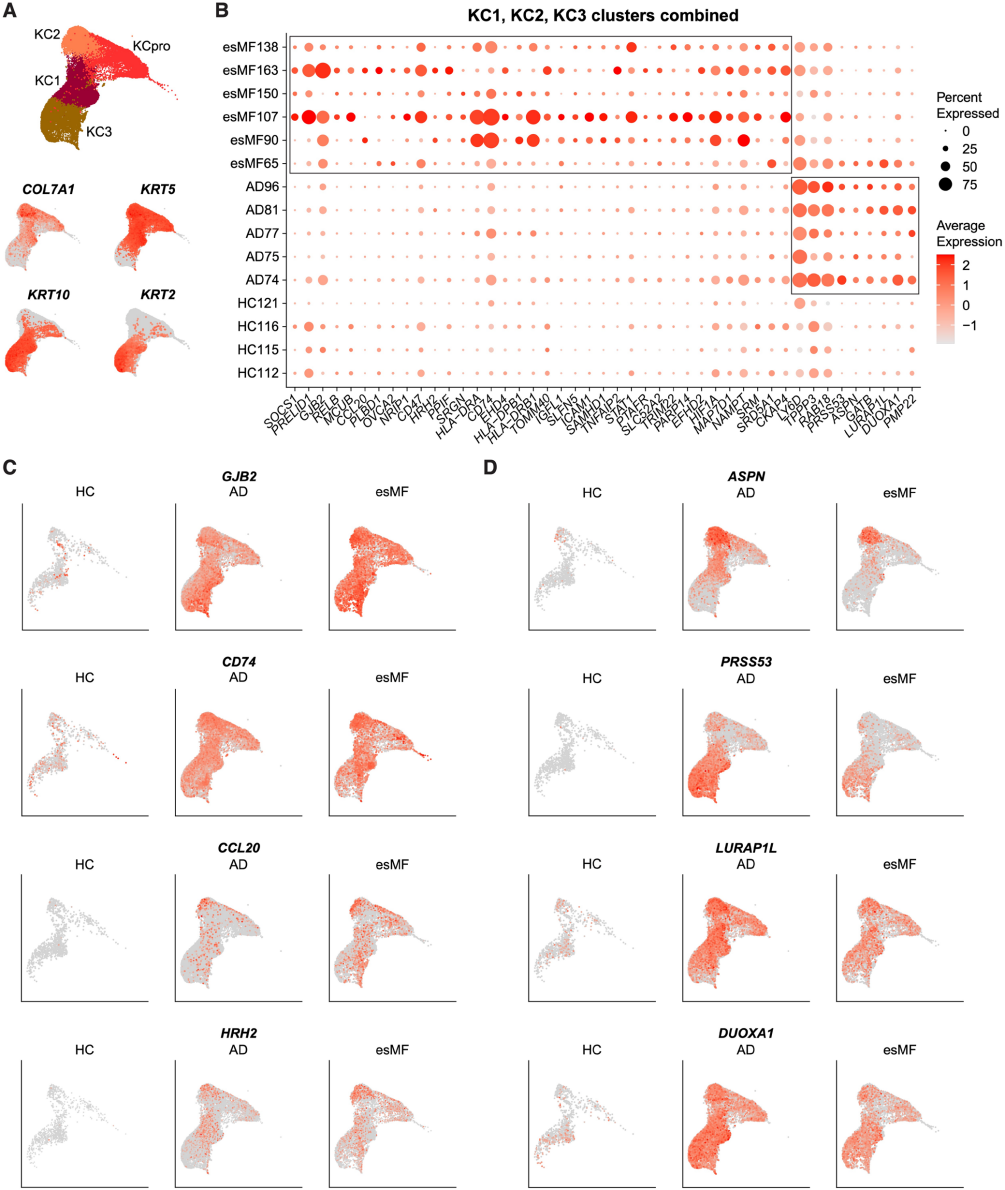
Keratinocyte genes differentiating esMF from AD lesions. (A) UMAP projections of all KC clusters and expression of canonical KC subset markers. (B) Dotplot of selected genes significantly and consistently upregulated in non‐proliferating KCs from esMF or AD patients. All genes displayed were selected from a list of DEGs with log2FC > 0.5 and *p*‐value < 0.05. (C) UMAP projections displaying expression of selected genes upregulated in KCs from esMF patients, split by disease group. (D) UMAP projections displaying expression of selected genes upregulated in KCs from AD patients, split by disease group.

Based on the prominence of DEGs observed in keratinocytes (KCs), we sought to further characterize the transcriptomic profile of these cells (Figure 3A). We defined four KC clusters: *KRT10+* suprabasal KC1, *COL7A1* + *KRT5*+ basal KC2, *KRT2* + *KRT10*+ suprabasal KC3, and a *MKI67+* proliferating cluster KCpro (Figures 3A and 1F). Combining non‐proliferating KCs, we next generated a list of genes that were specifically upregulated in either esMF or AD biopsies (Figure 3B). KCs from esMF samples strongly expressed genes related to type‐II interferon signalling (*SOCS1, STAT1, ICAM1*) and antigen presentation (*HLA‐DRA, HLA‐DPB1, HLA‐DRB1, CD74*). We also observed upregulated expression of gap junction beta‐2 protein/connexin 26 (*GJB2*) [13], the NAD+ biosynthesis gene nicotinamide phosphoribosyltransferase (*NAMPT*), and the hypoxia inducing factor *HIF1A* (Figure 3B,C). Several studies suggest that these three genes are upregulated during KC proliferation or may contribute to proliferative processes [14, 15, 16, 17]. Additionally, the CCR6 ligand *CCL20* was elevated in the KCs of several esMF patients (Figure 3C). *CCL20* expression is implicated in the KC response to injury and is associated with promoting metastasis of CTCL and other malignancies [18, 19]. We also observed upregulated expression of *HRH2* (histamine receptor H2) in KC of esMF samples (Figure 3C; Table S4). Histamine receptor H2 has been suggested to play a role in various skin functions, such as inhibiting skin barrier recovery and increasing KC proliferation [20].

In AD samples, KCs exhibited enhanced and selective expression of several genes including Lymphocyte Antigen 6 Family Member D (*LY6D*) [21], the extracellular matrix protein asporin (*ASPN*), and serine protease 53 (*PRSS53*) (Figure 3D). Additionally, elevated expression of leucine rich adapter protein 1‐like (*LURAP1L*) and dual oxidase maturation factor 1 (*DUOXA1*) was present in KCs from AD patients, both of which have been reported to be upregulated in response to IL‐13 signalling [3]. Together, these results demonstrate that KCs in esMF patients express genes related to type 1‐associated inflammation, proliferation, and tissue damage, possibly in response to CTCL‐mediated skin barrier perturbation.

### 3.4 Fibroblasts in esMF Patients Express Tumour‐Promoting Genes, Suggesting a Cancer‐Associated Fibroblast Phenotype

**FIGURE 4:**
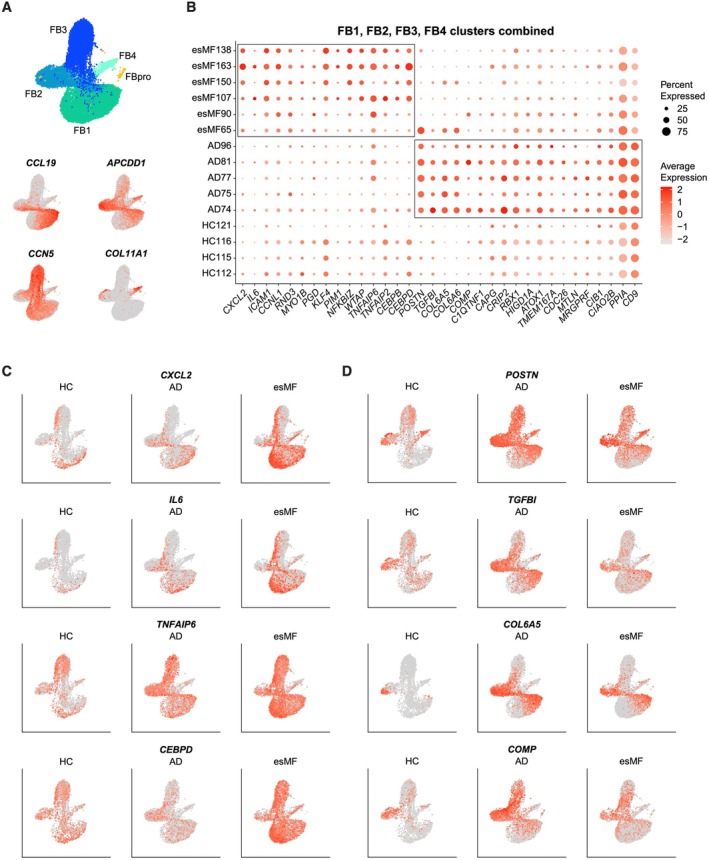
Fibroblast genes differentiating esMF from AD lesions. (A) UMAP projections of all FB clusters and expression of canonical FB subset markers. (B) Dotplot of selected genes significantly and consistently upregulated in non‐proliferating FBs from esMF or AD patients. All genes displayed were selected from a list of DEGs with log2FC > 0.5 and *p*‐value < 0.05. (C) UMAP projections displaying expression of selected genes upregulated in FBs from esMF patients, split by disease group. (D) UMAP projections displaying expression of selected genes upregulated in FBs from AD patients, split by disease group.

Recently, fibroblasts (FBs) have received significant attention in immuno‐oncology studies, particularly in the context of cancer‐associated fibroblasts (CAFs) [22, 23]. In our dataset, we found high numbers of DEGs within the FB population, both in esMF and AD (Figure 2A). Besides proliferating *MKI67+* cluster FBpro, we characterized four additional cell populations: cluster FB1 comprising *CCL19+* proinflammatory FBs, cluster FB2 with *APCDD1+* secretory papillary FBs, cluster FB3 with *CCN5+* secretory reticular FBs, and cluster FB4 that was composed of *COL11A1+* cartilage mesenchymal FBs (Figures 4A and 1F). When comparing a combination of non‐proliferative FB clusters of esMF vs. AD, the neutrophil homing marker *CXCL2* was one of the most upregulated genes in all FB clusters (Figure 4B,C). *CXCL2* expression has been described to have pro‐tumorigenic properties, and expression has been observed in CAFs [24]. *IL6*, which encodes for the proinflammatory cytokine interleukin‐6, was also strongly expressed in esMF FBs compared to those from AD and HC (Figure 4B,C). Interleukin‐6 is elevated in a broad array of cancer types and is predominantly thought to be associated with worse outcomes related to tumour progression in many carcinomas and hematologic malignancies [25]. We observed upregulation of the TNF‐responsive gene *TNFAIP6* in esMF (Figure 4C), which is reported to be expressed in CAFs and reduce anti‐tumour responses to immune checkpoint blockade therapy [26]. The genes encoding for the beta and delta versions of CCAAT enhancer‐binding proteins, *CEBPB* and *CEBPD*, were also upregulated in the FBs of esMF patients, the latter of which has been described to induce angiogenesis and metastasis in a breast cancer setting [27].

In FBs of AD patients, we observed strong expression of genes such as the extracellular matrix proteins periostin (*POSTN*) and transforming growth factor beta induced (*TGFBI*), as well as collagen type VI alpha 5 chain (*COL6A5*) (Figure 4D), all of which are implicated in the type 2 immune response associated with AD pathogenesis [28, 29, 30]. Furthermore, cartilage oligomeric matrix protein (*COMP*) was also upregulated in AD samples, which is known to contribute to collagen remodelling and tissue hardening [31]. Overall, our observations in FBs suggest that these cells exert a pro‐tumour gene program in esMF, while promoting hallmark type 2 immune responses in AD skin.

### 3.5 Dendritic Cells and Macrophages Express Genes Related to Suppression of Immune Activity in esMF

**FIGURE 5:**
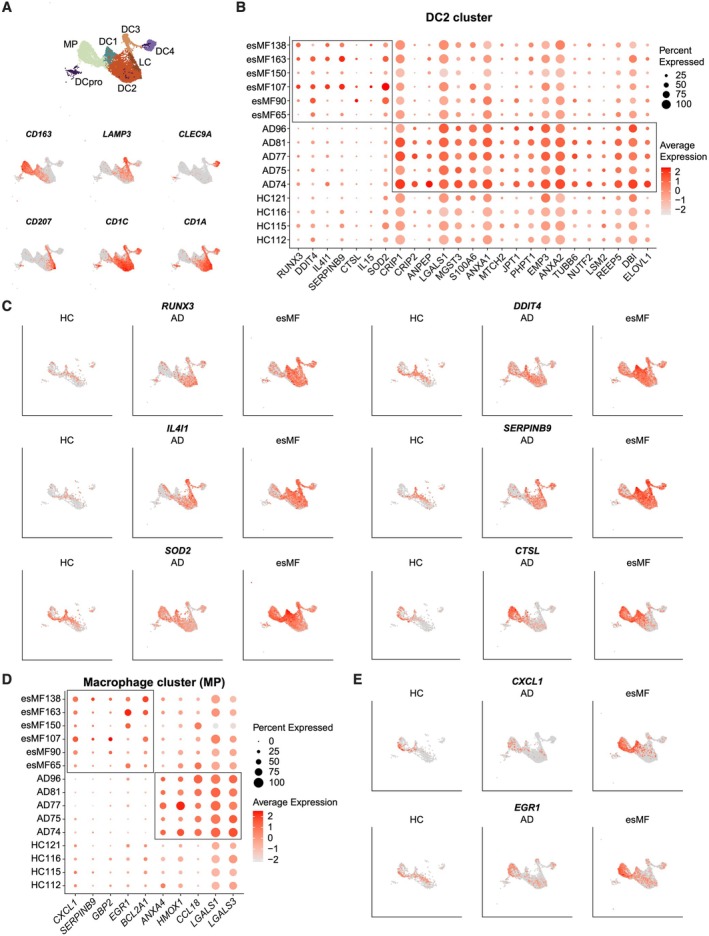
Myeloid cell genes differentiating esMF from AD lesions. (A) UMAP projections of myeloid cell types and canonical myeloid subset marker expression. (B) Dotplot of selected genes significantly and consistently upregulated in cluster DC2 from esMF or AD patients. All genes displayed were selected from a list of DEGs with log2FC > 0.5 and *p*‐value < 0.05. (C) UMAP projections displaying expression of selected genes upregulated in DC2 from esMF patients, split by disease group. (D) Dotplot of selected genes upregulated in cluster MP from esMF or AD patients. All genes displayed were selected from a list of DEGs with log2FC > 0.5 and *p*‐value < 0.05. (E) UMAP projections displaying expression of *CXCL1* and *EGR1* in cluster MP, split by disease group.

Myeloid cells are critical for bridging innate and adaptive immune responses [32]. We identified one *CD163+* macrophage cluster (MP), and six dendritic cell (DC) clusters: cluster DC1 (*CD1C* + *CD1A*‐), cluster DC2 (*CD1C* + *CD1A*+), cluster DC3 (*LAMP3*+), cluster DC4 (*CLEC9A*+), Langerhans cells (LC, *CD207*+), and proliferating *MKI67*+ DCpro (Figure 5A). Our analysis revealed a substantial amount of DEGs in several DC as well as the single macrophage (MP) cluster. Cluster DC2 upregulated several genes in esMF (Figure 5B), including RUNX family transcription factor 3 (*RUNX3*) and DNA damage inducible transcript 4 (*DDIT4*) (Figure 5C), both of which are reported to prevent DC immune activity and maturation, respectively [33, 34]. Interleukin 4 induced 1 (*IL4I1*) was also elevated in cluster DC2, which contributes to immunosuppression of T‐cells through metabolism of tryptophan, analogous to the behaviour of indoleamine‐pyrrole 2,3‐dioxygenase [35, 36, 37]. Upregulation of the antigen cross‐presentation gene *SERPINB9* in esMF samples was in line with studies demonstrating its elevated expression levels in various cancers [38, 39, 40]. We also measured elevated levels of superoxide dismutase 2 (*SOD2*), which is a reactive oxygen species scavenger that has demonstrated necessity for ample expression of MHC‐II, CD86 and CD44 in mice [41], and the lysosomal cysteine protease cathepsin L (*CTSL*) (Figure 5C).

MP in esMF upregulated the chemokine *CXCL1* (Figure 5D,E), which is reportedly expressed in tumour‐associated macrophages (TAMs) [42]. Like in DCs, *SERPINB9* was upregulated in the MP of esMF skin. We also observed elevated expression of the transcription factor early growth factor 1 (*EGR1*) in esMF MP, which is important for regulating transcription in a variety of cell types. In AD, MP expressed *CCL18* (Figure 5D), which is known to be upregulated in this disease compared to healthy or psoriatic skin [43]. Overall, these results suggest that myeloid cells in esMF lesions express an immunomodulatory transcriptional program, which may enable the malignant progression of MF.

### 3.6 Subpopulations of T‐Cells Express Exhaustion‐Associated Genes in esMF, Suggesting Impaired Defence Mechanisms Against Malignant Cells

**FIGURE 6:**
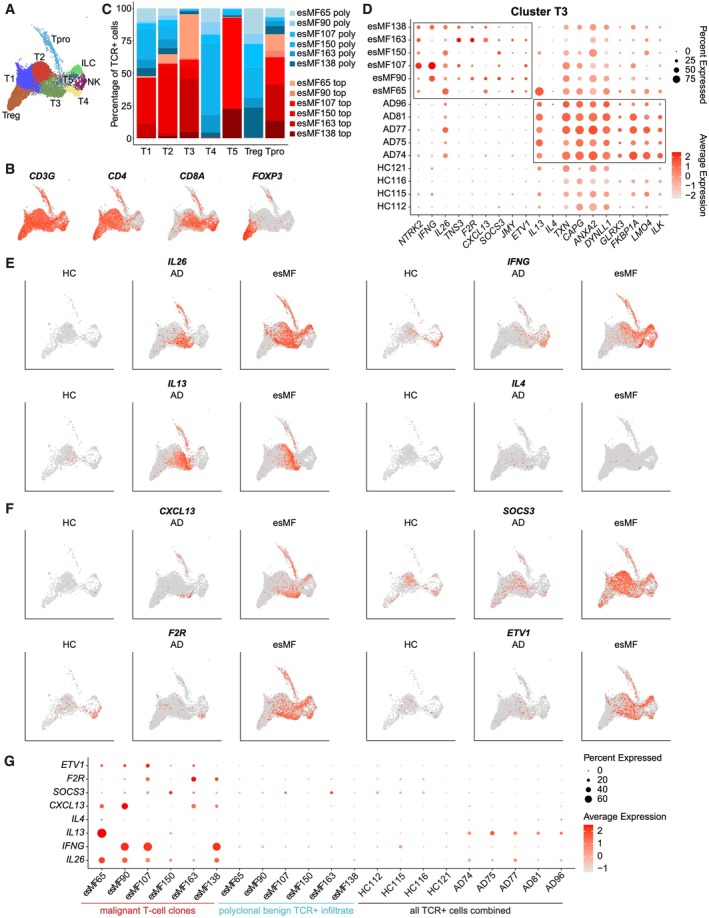
T‐cell genes differentiating esMF from AD lesions. (A) UMAP projection of lymphocyte subsets. (B) UMAP projections of T‐cell lineage markers across lymphocyte subsets. (C) Barplot showing patient and TCR clone contribution to each T‐cell subset. (D) Dotplot of selected genes significantly and consistently upregulated in cluster T3 from esMF or AD patients. All genes displayed were selected from a list of DEGs with log2FC > 0.5 and *p*‐value < 0.05. (E) UMAP projections displaying expression of selected genes previously described to be upregulated in esMF or AD, split by disease group. (F) UMAP projections displaying expression of selected genes upregulated in T3 from esMF patients, split by disease group. (G) Dotplot showing expression of selected genes in top malignant T‐cell clones from esMF samples, polyclonal T‐cells from esMF samples, and total T cells from HC or AD samples.

Since MF is a T‐cell‐mediated disease, it was imperative to assess gene expression changes within the T cell compartment. Excluding B‐cells, clustering analysis of lymphocytes revealed one NK‐cell cluster, one ILC‐like cluster, and seven T‐cell clusters: cluster T1 was predominantly *CD4*+ helper T‐cells, and cluster T2 and T3 had mixed expression of *CD4* and *CD8A*, with cluster T3 being > 90% dominated by CD4+ malignant T‐cell clones. Cluster T4 was predominantly *CD8*+ cytotoxic T‐cells comprised of > 90% polyclonal T‐cell receptor‐expressing cells (Figures 1F and 6A–C). Cluster T5 expressed a mix of *CD4+* and *CD8A+* and was > 50% dominated by the top clone of patient esMF107, cluster Treg was *FOXP3*+ regulatory T‐cells, and cluster Tpro was *MKI67+* proliferating T‐cells comprised of > 75% esMF top clones (Figure 6A–C). Importantly, cluster T3 harboured the highest number of upregulated DEGs when comparing esMF to AD skin (Figure 2A), including several pro‐inflammatory mediators previously detected in CTCL lesions such as *IL26* [44] and *IFNG* [5, 45, 46] (Figure 6D,E). Conversely, we observed strong expression of the Th2‐hallmark cytokine *IL13* in AD samples [3, 29] (Figure 6D,E). IL‐13 has been shown to be overexpressed in CTCL but to a lesser extent than in AD [47]. Although expression of *IL13* and *IL4* are both implicated in AD [3], *IL4* expression was detected in only a few cells across all samples, in line with previous reports [48, 49]. In T‐cells of esMF skin, we additionally measured upregulation of the B‐cell attracting chemokine *CXCL13*, suppressor of cytokine signalling 3 (*SOCS3*), coagulation factor II thrombin receptor (*F2R*), and ETS variant transcription factor 1 (*ETV1*) (Figure 6F), all of which have been associated with T‐cell exhaustion or poor immune infiltration and response against cancer [50, 51, 52, 53]. Further analyses revealed that these selected genes were produced predominantly in malignant clones of the esMF T‐cell pool, expressing the dominant T‐cell receptor clone in each patient sample, as opposed to the polyclonal (i.e., reactive) immune infiltrate (Figure 6G). In contrast, both polyclonal T‐cells from esMF samples and all TCR+ T‐cells from HC and AD samples had little to no expression of most of the selected markers, with the AD hallmark cytokine *IL13* being the exception (Figure 6G).

## 4 Discussion

Proper diagnosis of esMF remains a challenge due to the clinical and histopathological similarities between this malignancy and benign inflammatory skin conditions such as AD. Although it is well established that a strongly expanded T‐cell clone in a given skin disease preferentially classifies it as CTCL [6], additional understanding of the malignancy's defining features are urgently needed. Using single‐cell RNA sequencing data from esMF, in comparison to AD or HC skin biopsies, we observed esMF‐specific upregulation of genes in various cell types from both stromal and immune lineages. While previous studies have often primarily investigated T‐cell intrinsic differences in esMF [54, 55], we focused on gene expression changes that occurred in the KC, FB, and myeloid compartments of the stroma, given their strong differences in gene expression from HC samples.

KCs expressed a plethora of genes demonstrating response to interferon (specifically IFN‐γ signalling). In line, we observed upregulation of antigen presentation genes in the KCs of esMF samples, which is well known to occur following IFN‐γ stimulation [56, 57]. This is concurrent with our result that T‐cells in several esMF samples expressed elevated levels of *IFNG*. Some of the other top upregulated genes (*GJB2*, *NAMPT*, *HIF1A*) in esMF KC have reported associations with KC proliferation [14, 15, 16, 17]. Connexin 26 (*GJB2*) is a gap junction protein that is elevated in hyperproliferative epidermis and can block healing and remodelling of wounded skin while retaining large portions of the immune infiltrate [58]. Inhibition of the nicotinamide phosphoribosyltransferase (gene *NAMPT*) was demonstrated in zebrafish models to prevent both KC hyperproliferation and cell death, underlining the role of *NAMPT* in these KC functions [16]. The transcription factor hypoxia‐inducible factor 1‐alpha (gene HIF1A) controls expression of a broad array of proteins, several of which have been reported to enhance KC proliferation: ECE1, WAF1, and CIP1 [14, 15]. Therefore, upregulation of *HIF1A* expression may indirectly contribute to KC proliferation through activation of several gene programs. Together, these results reveal that KC in esMF patients show a signature of interferon signalling, which may consequently increase expression of antigen presentation and proliferation‐associated genes.

Our data in esMF FBs revealed upregulation of genes associated with tumorigenesis: *CXCL2*, *IL6*, *TNFAIP6*, and *CEBPD*. This suggests that the FBs in esMF samples exert a CAF phenotype. *CXCL2* has been found to be upregulated in colorectal, liver, and gastric cancers, while also directly correlating with disease progression in lung cancer [24]. The CXCL2‐CXCR2 signalling axis can activate several growth‐associated pathways such as ERK/MAPK and PI3K/AKT, while also promoting recruitment of myeloid‐derived suppressor cells and neutrophils to tumour sites [59, 60]. Interestingly, we observed no neutrophils in our esMF skin biopsies, despite elevated *CXCL2* expression. Murine studies of chronic wounds have demonstrated that elevated *CXCL2* expression can recruit macrophages with no effect on neutrophil infiltration levels in the skin [61]. Thus, it is possible that the chronic nature of esMF lesions is characterized by a similar lack of neutrophils. Upregulation of the pro‐inflammatory cytokine *IL6* mirrored previous observations in head and neck, pancreatic, non‐small cell lung, and breast cancers [25]. Furthermore, IL‐6 signals through STAT3, which was demonstrated to promote expression of cell cycle and anti‐apoptotic genes in cancer cells [62]. Some CTCL patients also exhibited elevated levels of IL‐6 [63], highlighting its potential significance in this disease context. We additionally observed upregulation of *TNFAIP6* (encoding for TSG‐6), which is stimulated by TNF and its expression in FBs has been shown to inhibit checkpoint blockade therapy in pancreatic cancer patients [26]. *CEBPD* upregulation in esMF FBs further suggests CAF‐like behaviour, as CEBPD expression in FBs can promote angiogenesis by inducing secretion of stromal‐cell‐derived factor 4 into the tumour microenvironment [27]. The overall gene expression profile observed in the FBs of esMF patients is indicative of a tumour‐supportive phenotype, with genes encoding for several secreted factors (*CXCL2*, *IL6*). Taken together, the pro‐tumour gene signature found in esMF FBs may facilitate future investigation of therapeutic targets for CTCL and other haematological malignancies.

Like the FBs in esMF samples, DCs and MP exhibited a gene expression program characteristic of supporting malignant progression by restraining anti‐tumour immune responses. We found DCs in esMF skin to upregulate many genes that reportedly inhibit their maturation and overall immune function. *RUNX3* was shown to dictate the immunosuppressive activity of TGF‐β in DCs, with ablation of this gene in mice desensitizing DCs to TGF‐β exposure [33]. Following *Runx3* knockout in mice, TGF‐β treated DCs were able to upregulate MHC‐II/CD86 expression associated with maturation, while wild‐type DCs were unable to, due to TGF‐β‐mediated suppression [64]. *DDIT4* expression inhibits mTORC1 activity, which is critical for DC maturation [65]. The tryptophan metabolizer interleukin‐4 induced gene 1 (*IL4I1*) functions in a similar manner to IDO, a well‐studied oxidase that is elevated in many solid and hematologic tumors [36, 37]. IDO has immunosuppressive roles not only in the context of cancer but also in organ transplantation and protection of the placenta during pregnancy [66, 67, 68]. Characterization of the IL4I1 protein revealed that its production in DCs inhibits T‐cell proliferation and decreases CD3ζ‐chain (*CD247*) expression, which is required to license T‐cell activation and cytotoxicity [35]. Finally, heterozygous expression of *SOD2* in mice was demonstrated to reduce DC expression of MHC‐II, CD86, and CD44, which are critical for antigen‐presentation and T‐cell priming functions [41]. Notably, while we observed upregulation of several HLA genes in the KCs of esMF, none of these genes were significantly upregulated in DCs of esMF, suggesting a possible impairment in antigen presenting capacity of these DCs.

MP in esMF upregulated several genes that are associated with a TAM profile, which have been reported to be present in CTCL [69]. *CXCL1* expression in TAMs was demonstrated to promote breast cancer metastasis [42]. Additionally, blocking of CXCL1 interactions with its cognate receptor (CXCR2) was shown to sensitize hepatocellular carcinoma to chemotherapy [70], suggesting a pro‐tumorigenic role of this signalling pathway. *EGR1* upregulation also suggests a tumour supportive phenotype of these MP, as it has been described to suppress macrophage inflammatory responses and is upregulated after exposure to M2‐stimulatory cytokines [71, 72, 73]. Our data in esMF‐derived myeloid cells largely present a phenotype that is representative of a blunted immune response. Consequently, both DCs and MP may intrinsically have reduced activity against malignant cells while also exhibiting impaired capability to licence cells of the adaptive immune system.

T‐cells in esMF lesions upregulated several genes previously reported to be elevated in CTCL. *IFNG* expression has been shown to be elevated in esMF and to decline with disease progression [74]. Additionally, *IL26* is elevated in CTCL samples [75, 76]. Other genes expressed in esMF‐derived T‐cells have previously been associated with T‐cell exhaustion: *CXCL13*, *SOCS3*, *F2R*, and *ETV1*. In fact, *CXCL13* was predominantly expressed in T‐cell clusters that were either proliferating or dominated by a top malignant clone. CXCL13 is a chemokine previously described to be elevated both generally in CTCL and more specifically in Sezary syndrome [55, 77, 78]. *SOCS3* upregulation was in line with the elevated *IL6* expression by FBs, as IL‐6 signalling is known to induce expression of *SOCS3* as a feedback inhibition mechanism [79]. Although the role of IL‐6 signalling on T‐cell behaviour is complex, *SOCS3* deletion has conferred enhanced anti‐tumour activity to T‐cells [51]. While the Th2 cytokine *IL13* was consistently elevated in AD samples, we measured only weak *IL4* expression across all samples in this study, in line with previous reports of human skin [80, 81]. Both these and our data may support the idea that IL‐13 exerts a stronger contribution to AD than the classic Th2 cytokine IL‐4.

This study aimed to uncover transcriptomic differences in various cell types, to better understand the behaviours and characteristics of the distinct esMF and AD pathologies. Still, there are several limitations to this study. Despite the ability of scRNA‐seq to profile thousands of cells within a single biopsy, we obtained an overall small sample size of six esMF patients and five AD patients. While our findings demonstrate consistent patterns observed across most patients in respective disease groups, larger cohorts will be needed to validate the observations we present in this study, particularly for purposes of diagnostic biomarker development. Another limitation to consider is the overlap of the esMF inflammatory gene signature with other skin dermatoses that were not investigated in this study, such as psoriasis. Nonetheless, we did not find the strong Th17 response in our esMF samples that is typically found in psoriasis [82], which might be a major distinguishing feature. However, the interferon response, especially the type‐I interferon signalling that we found in our esMF samples, is also typically present in psoriasis, and thus needs further investigation.

Taken together, our data present an esMF‐driven transcriptomic profile of inflammation and proliferation in KCs, pro‐tumour molecular programs in FBs and myeloid cells, combined with an exhausted gene expression profile in T‐cells. Considering our results in FBs and myeloid cells, it is plausible that the immunomodulatory gene expression profile of these subsets may contribute to an exhaustive or impaired T‐cell phenotype in esMF lesions. These data also present gene expression distinctions that may be leveraged as biomarkers for future diagnostic and potentially therapeutic purposes.

## Author Contributions

B.D. performed the formal analysis, created the figures, and wrote the manuscript; C.W. helped with data curation and project administration, N.A. and A.K. supported the methodology and code components. C.J. helped with conceptualization. P.M.B. secured funding and supervised the project. All authors reviewed, edited, and approved the final manuscript.

## Funding

This work was supported in part through the National Institute of Arthritis and Musculoskeletal and Skin Diseases award # R01AR083960. This work was supported in part through the computational and data resources and staff expertise provided by Scientific Computing and Data at the Icahn School of Medicine at Mount Sinai and supported by the Clinical and Translational Science Awards (CTSA) grant UL1TR004419 from the National Center for Advancing Translational Sciences. Research reported in this publication was also supported by the Office of Research Infrastructure of the National Institutes of Health under award number S10OD030463. The content is solely the responsibility of the authors and does not necessarily represent the official views of the National Institutes of Health.

## Conflicts of Interest

C.J. has received personal fees from LEO Pharma, Pfizer, Kyowa Kirin, UCB Pharma, Boehringer Ingelheim, BMS, Recordati Rare Diseases, Eli Lilly, Novartis, Takeda, Stemline Therapeutics, AbbVie, Janssen, STADA, and Almirall. C.J. is an investigator for Almirall, Janssen, AbbVie, and Incyte. P.M.B. has received personal fees from Almirall, Sanofi, Janssen, LEO Pharma, AbbVie, Pfizer, Boehringer Ingelheim, GSK, Regeneron, Eli Lilly, Celgene, Novartis, UCB, Merck, RAPT Therapeutics, Galderma, Immunocore, TD Securities, Apogee, and Bristol Myers Squibb. P.M.B. has received research support from Pfizer (grant paid to his institution) and is an investigator for Pfizer and AbbVie. The rest of the authors declare that they have no relevant conflicts of interest.

## Supporting information


**Table S1:** Patient baseline characteristics at time of sampling. TCS topical corticosteroids, TCI topical calcineurin inhibitors, NB‐UVB narrow band ultraviolet B, PUVA psoralen ultraviolet A, UV ultraviolet, n.a. not applicable.

**Table S2:** Cell counts per patient and cell cluster after quality control (QC) filtering. AD = atopic dermatitis, esMF mycosis fungoides, HC = healty control.

**Table S3:** Top 50 significantly expressed genes in each cell cluster, compared to all other clusters. The top 50 differentially expressed genes from each cell cluster compared to all other cell clusters were ordered by log2FC, omitting genes with *p*‐value ≥ 0.05. T: T‐cells, Tpro: proliferating T cells, NK: natural killer cells, ILC: innate lymphoid cells, Bcells: B cells, LC: Langerhans cells, DC: dendritic cells, pDC: plasmacytoid dendritic cells, MP: macrophages, MC: mast cells, Neronal: neuronal cells, MEL: melanocytes, SMC: smooth muscle cells, LEC: lymphatic endothelial cells, BEC: blood endothelial cells, Peri: pericytes, FB: fibroblasts, SwG: sweat gland cells KC: keratinocytes.

**Table S4:** Pairwise comparisons of differentially expressed genes in each cell cluster. Differentially expressed genes were filterted for *p*‐value < 0.05 and then ordered by log2FC for each pairwise comparison: AD vs. HC, esMF vs. HC, and esMF vs. AD.

## Data Availability

The data that support the findings of this study are openly available in Gene Expression Omnibus at https://www.ncbi.nlm.nih.gov/geo/, reference number GSE247047.
