# Supplementary material for: Molecular Markers Distinguishing Early‐Stage Mycosis Fungoides From Atopic Dermatitis Skin Lesions
Source: Exp Dermatol. 2026 Mar 26;35(4):e70240. doi: 10.1111/exd.70240 (PMC13022068; doi:10.1111/exd.70240)
Supplement: Supplementary file 1 — Table S1: Patient baseline characteristics at time of sampling. TCS topical corticosteroids, TCI topical calcineurin inhibitors, NB‐UVB narrow band ultraviolet B, PUVA psoralen ultraviolet A, UV ultraviolet, n.a. not applicable. [file EXD-35-e70240-s004.docx]

| **Subject ID** | **Diagnosis after clnicopathological correlation** | **Age** | **Sex** | **Race** | **Disease duration (yrs)** | **Specific treatment at sampling** | **Previous treatments** | **Disease stage** |
| --- | --- | --- | --- | --- | --- | --- | --- | --- |
| 112 – HC | Healthy control | 51 | F | White | n.a. | n.a. | n.a. | n.a. |
| 115 – HC | Healthy control | 48 | M | White | n.a. | n.a. | n.a. | n.a. |
| 116 – HC | Healthy control | 56 | F | White | n.a. | n.a. | n.a. | n.a. |
| 121 – HC | Healthy control | 44 | F | White | n.a. | n.a. | n.a. | n.a. |
|  |  |  |  |  |  |  |  |  |
| 74 – AD | Atopic dermatitis | 26 | M | White | 24 | None | TCS, TCI | n.a. |
| 75 – AD | Atopic dermatitis | 24 | F | White | 10 | None | TCS, Phototherapy (NB-UVB) | n.a. |
| 77 – AD | Atopic dermatitis | 26 | M | White | 12 | None | TCS, Phototherapy (NB-UVB) | n.a. |
| 81 – AD | Atopic dermatitis | 53 | M | White | 50 | None | TCS, TCI, Phototherapy (NB-UVB) | n.a. |
| 96 – AD | Atopic dermatitis | 51 | M | White | Since childhood | None | TCS | n.a. |
|  |  |  |  |  |  |  |  |  |
| 65 – esMF | Early-stage mycosis fungoides | 53 | M | White | 10 | none | TCS | IB (T2bN0M0B0) |
| 90 – esMF | Early-stage mycosis fungoides | 75 | M | White | 11 | none | TCS, Phototherapy (PUVA, NB-UVB), bexarotene, acitretin | IB (T2aN0M0B0) |
| 107 – esMF | Early-stage mycosis fungoides | 39 | F | White | 22 | none | TCS | IA (T1bNxMxB0) |
| 150 – esMF | Early-stage mycosis fungoides | 56 | M | White | 12 | none | TCS, Phototherapy (PUVA, NB-UVB), natural UV- exposure | IA (T1bNxMxB0) |
| 163 – esMF | Early-stage mycosis fungoides | 83 | F | White | 3 | none | TCS, Phototherapy (NB-UVB), natural UV exposure | IB (T2aNxMxB0) |
| 138 – esMF | Early-stage mycosis fungoides | 47 | M | White | 1 | none | TCS | IA (T1aNxMxB0) |

**Table S1.** Patient baseline characteristics at time of sampling; *n.a. not applicable, TCS topical corticosteroids, TCI topical calcineurin inhibitors, NB-UVB narrow band ultraviolet B, PUVA psoralen ultraviolet A, UV ultraviolet*
